# Audit of the STOMP Pathway and Its Impact on Reducing Psychotropic Prescribing in People With Learning Disabilities

**DOI:** 10.1192/bjo.2026.11837

**Published:** 2026-06-30

**Authors:** Alam Shah, Chinwe Vivian Okeke, Judith Gee

**Affiliations:** 1 CNTW NHS Foundation Trust, Newcastle, United Kingdom; 2 tevs nhs foundation trust, middlesbrough, United Kingdom

## Abstract

**Aims:**

To assess the effectiveness of the STOMP pathway in supporting the reduction of psychotropic medication prescribing among people with learning disabilities.

**Methods:**

Adults with learning disabilities under the care of the Community Learning Disability Team who were prescribed one or more psychotropic medications were considered. Reviewed data from January 2024 to April 2025 and sources included Electronic health records, STOMP review templates, MDT notes. There were 30 participants in the sample.

**Results:**

The audit demonstrated a high level of compliance across most criteria relating to psychotropic medication use. Documentation of the clinical rationale for psychotropic useachieved full compliance (100%). Medication reviews within the previous 12 months were completed for the majority of individuals (93%). Non-pharmacological interventions were documented in 87% of cases, indicating good but not universal adherence. Evidence of involvement of the person and/or their carer in decision-making was present in 90% of records. However, documentation of attempted or completed reduction in psychotropic medication was comparatively lower (70%), highlighting an area for potential improvement.

**Conclusion:**

The audit shows that the STOMP pathway has had a **positive impact on reducing unnecessary psychotropic prescribing** in people with learning disabilities. Continued focus on structured reviews, non-drug alternatives, and stakeholder involvement will help sustain this progress.

